# Bioinformatical parsing of folding-on-binding proteins reveals their compositional and evolutionary sequence design

**DOI:** 10.1038/srep18586

**Published:** 2015-12-18

**Authors:** Mohanalakshmi Narasumani, Paul M Harrison

**Affiliations:** 1 Department of Biology, McGill University, Montreal, QC, Canada

## Abstract

Intrinsic disorder occurs when (part of) a protein remains unfolded during normal functioning. Intrinsically-disordered regions can contain segments that ‘fold on binding’ to another molecule. Here, we perform bioinformatical parsing of human ‘folding-on-binding’ (FB) proteins, into four subsets: Ordered regions, FB regions, Disordered regions that surround FB regions (‘Disordered-around-FB’), and Other-Disordered regions. We examined the composition and evolutionary behaviour (across vertebrate orthologs) of these subsets. From a convergence of three separate analyses, we find that for hydrophobicity, Ordered regions segregate from the other subsets, but the Ordered and FB regions group together as highly conserved, and the Disordered-around-FB and Other-Disordered regions as less conserved (with a lesser significant difference between Ordered and FB regions). FB regions are highly-conserved with net positive charge, whereas Disordered-around-FB have net negative charge and are relatively less hydrophobic than FB regions. Indeed, these Disordered-around-FB regions are excessively hydrophilic compared to other disordered regions generally. We describe how our results point towards a possible compositionally-based steering mechanism of folding-on-binding.

Intrinsically disordered regions, in at least one of their functional modes, do not have a well-defined three-dimensional structure under physiological conditions^1^. They are involved in specific functions such as molecular recognition, molecular assembly, protein modification, and entropic chain activities^2^. They are mostly found in eukaryotes rather than in prokaryotes^3^,^4^. Approximately a third of proteins in eukaryotes are estimated to contain long disordered regions with 30 amino acids or higher^3^,^5^. These regions are associated with a wide variety of functions, most notably signal transduction, transcription and translation regulation^3^,^5^. Disordered regions are characterised by using several approaches, such as analysis of areas with missing electron density in an X-ray determined structure, or by NMR spectroscopy. They can be predicted by algorithms that analyze charge, hydrophobicity, low sequence complexity, amino acid composition and other factors^6^,^7^,^8^,^9^. Statistical studies of amino acid sequences in disordered regions show that they are significantly different than ordered regions^10^.

Protein interaction analysis has showed that disordered regions are abundant in proteins with large numbers of interacting partners^11^,^12^. Many proteins with disordered regions exhibit coupled folding and binding which has been proved to be a common process of molecular recognition and plays significant roles in protein function^13^,^14^. Such disordered regions, which are termed here ‘folding on binding’ (FB) regions, are highly flexible and exhibit a well-defined structure only upon binding to a specific partner molecule^15^. These regions have been reported to confer high specificity towards a partner molecule^16^.

In general, disordered regions are usually characterised by low hydrophobicity and somewhat higher net charge^17^,^18^. However, such trends are not clear for the specific character of FB regions^19^,^20^. A study of FB region complexes showed that the interfaces of FB regions are enriched in hydrophobic residues and appear to be more conserved than other disordered regions in the same proteins^21^. A comparative study on the evolution of ordered and disordered proteins suggested that disordered proteins evolve more rapidly than ordered proteins^17^. However, this condition is not always true and also a smaller group of disordered proteins appear to evolve very slowly^22^. Analysis of the evolution of disordered regions has thus yielded contradicting results^23^,^24^.

Here, we have studied the composition and conservation of proteins that form FB regions in human protein complexes. Specifically, we have parsed these proteins into four subsets of sequence: (i) Ordered regions, (ii) FB regions, (iii) disordered regions around FB regions (‘Disordered-around-FB’), and (iv) Other-Disordered regions in the proteins. We wish to ask whether the composition, and conservation behaviour across eukaryotic orthologs for these proteins is significantly different for these biophysically relevant subsets. We found a complex pattern of conservation and composition, with all of these regions having significantly different combinations of composition and conservation behaviour. Indeed, ‘Disordered-around-FB’ regions are the least hydrophobic regions, and more evolutionarily variable, and the FB regions are of comparable hydrophobicity to Other-Disordered regions in the proteins. We discuss the mechanistic implications of this compositional sequence design.

## Results and Discussion

### Overview of the data sets

**Figure 1:**
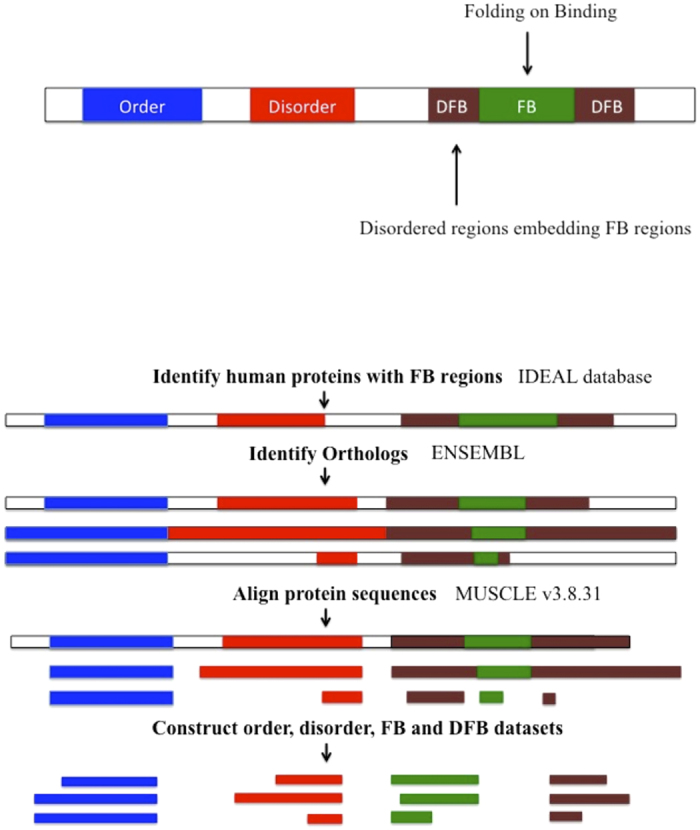
Pipeline of the analysis performed. Regions are classified as FB if they are ever labelled as folding-on-binding in the IDEAL database. Disordered-around-FB regions are the parts of disordered regions that embed these FBs (and which have never been detected as FBs themselves).

**Figure 2:**
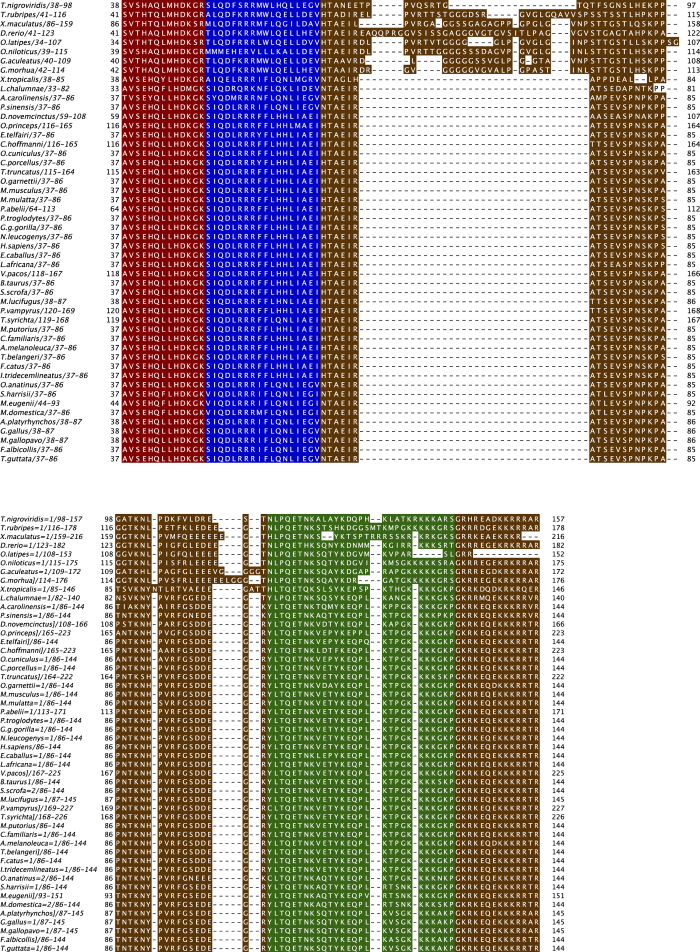
Example of a parsed protein. Multiple sequence alignment of human parathyroid hormone-like protein and its vertebrate orthologs, depicted using JalView^44^, showing the four region types. This figure uses the same colour scheme as Fig. 1.

From the 99 human proteins containing FB regions that are the subject of this study (Suppl. Table 1), were parsed the following four sets of regions: (i) ’Ordered’ protein domains; (ii) folding-on-binding regions (‘FB’ set); (iii) the intrinsically-disordered regions around FB regions (‘Disordered-around-FB’ regions), and (iv) intrinsically disordered regions that do not contain FB regions (‘Other-Disordered’ regions). The Ordered region set comprises experimentally verified structures that do not have a known alternative intrinsically-disordered state. The Disordered-around-FB and Other-Disordered regions are only experimentally reported as intrinsically disordered. The FB regions contain experimentally determined structure in bound form to their partner molecule, as well as being shown to be intrinsically disordered at other times. These data sets are compared for their trends in composition and conservation, as populations of sequences, using the pipeline of methods detailed in Fig. 1. The conservation of the four parsed region types across vertebrate evolution was analyzed, and a conservation score calculated (as detailed in Methods). An example of the parsing of a sequence into the four subsets is shown for human parathyroid hormone –like protein (Fig. 2), with the same colour scheme as Fig. 1.

### Analysis of Ordered, FB, Disordered-around-FB and Other-Disordered regions as populations of sequences

**Figure 3:**
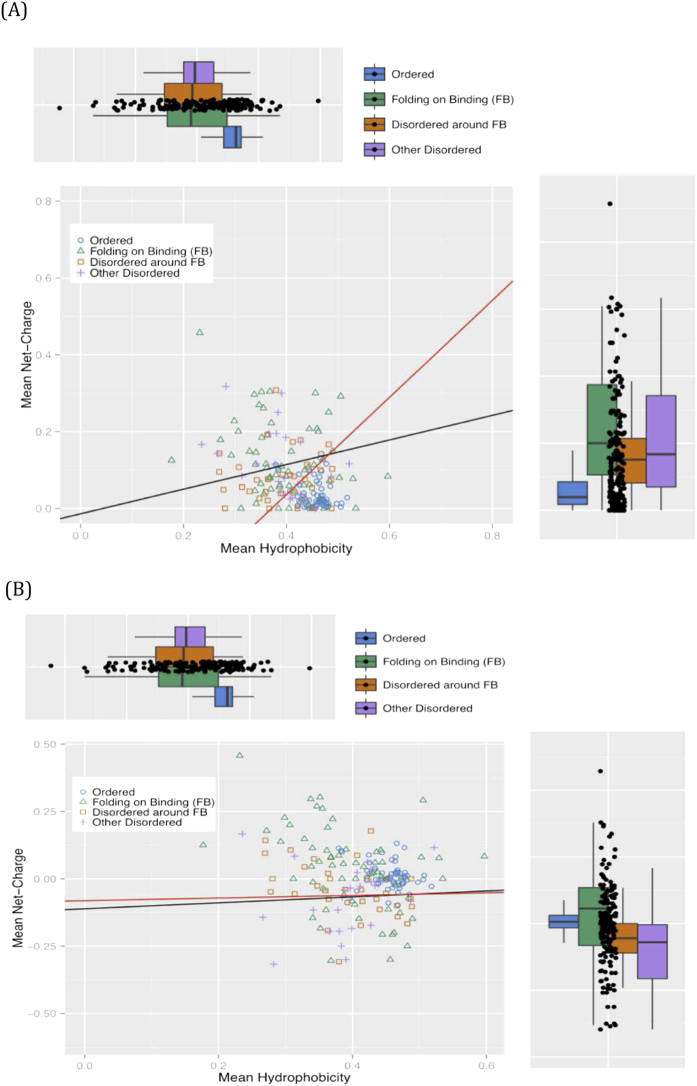
Analysis of the four region types as populations of sequences. Only fragments ≥20 residues in length are used in the plots. The values of mean hydrophobicity and mean conservation score are normalized to the range [0, 1]. (**A**) Mean hydrophobicity versus mean net-charge (absolute value). Lines were fitted to discriminate between Ordered and Other-Disordered regions by iterative Monte Carlo sampling of a wide range of intercept and slope values. The two lines (red and black) represent the two extremes of slope that give the same best percentage discrimination of Ordered regions (100%) (equations C = 1.21 H – 0.34, and C = 0.47 H – 0.06, where C is the mean net charge and H is the mean hydrophobicity, in the fragments). Here the absolute value of the mean net-charge is used (i.e., negative values are made positive). Box plots are drawn using the same colour coding as the main scatter plot. The whiskers extend from the hinge to the highest/lowest values that are within 1.5 * IQR of the hinge, where IQR is the inter-quartile range, or distance between the first and third quartiles. (**B**) Mean hydrophobicity versus mean net-charge (raw value). Lines were fitted as above in (**A**). The two lines (red and black) represent the two extremes of slope that give the same best percentage discrimination of Ordered regions (94%) (equations C = 0.11 H – 0.11, and C = 0.05 H – 0.08, where C is the mean net charge and H is the mean hydrophobicity, in the fragments).

Firstly, we asked whether we could distinguish the four region types according to their broad compositional characteristics. Comparison of mean hydrophobicity and mean net charge of the four parsed region types is shown in Fig. 3A,B. For the first plot, we use the absolute value of the mean net charge (Fig. 3A), and for the second plot the raw mean net charge value (Fig. 3B; see *Methods* for details). In these plots we only consider longer tracts, ≥20 residues. In line with a previous study^18^, the Ordered subset stands out as more hydrophobic than the three other region types. We fitted lines (as described in the figure legend) that give us optimum discrimination (>95%) of the Ordered subset from the Other-Disordered set. The black and red represent the two extremes of slope for such fitted boundary lines (Fig. 3A). In Fig. 3A, the other three sets scatter on either side of the lines and are not well segregated (24%–46% on the other side of the line). In Fig. 3B, using the raw value of the mean net charge, while the two disordered sets are not well discriminated from the Ordered set (39–50%), the FB regions segregate better with the Ordered set (74% on same side of the line).

**Figure 4:**
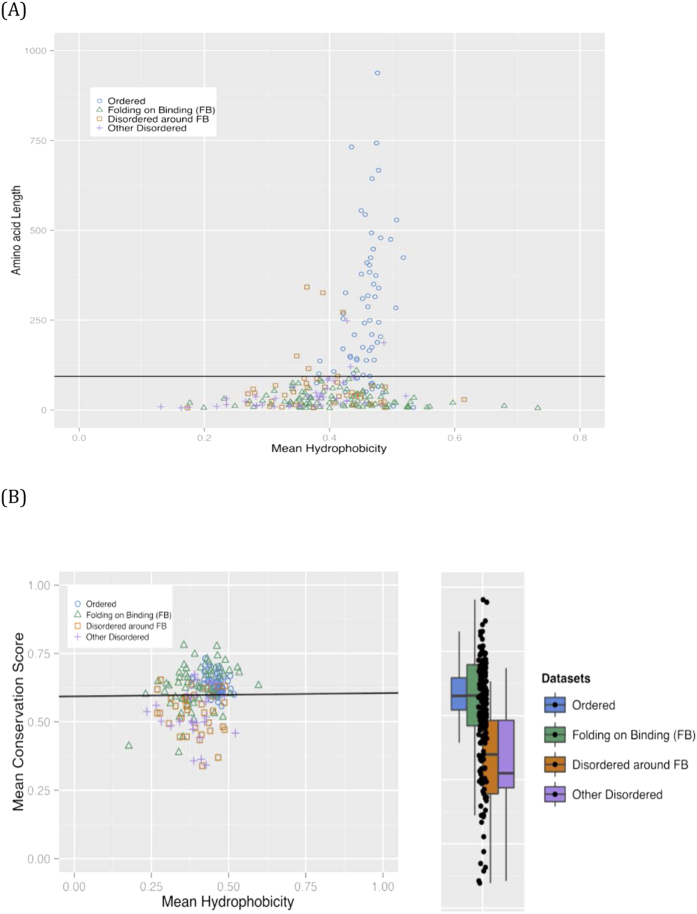
Analysis of the four region types as populations of sequences. Only fragments ≥20 residues in length are used in the plots. The values of mean hydrophobicity and mean conservation score are normalized to the range [0,1]. (**A**) Mean Hydrophobicity versus length. The colour scheme is as for Fig. 3. A simple length threshold of *region length = 93* was found to be the best boundary between Ordered and Other-Disordered regions; the same line was also optimal for discriminating between Ordered and either Disordered-around-FB or FB regions. (**B**) Mean conservation score versus mean hydrophobicity. The colour scheme is as for part (**A**). An almost horizontal line was found to be the best boundary between Ordered and Other-Disordered regions (equation S = 0.01 H + 0.59, where S is the mean conservation score and H is the mean hydrophobicity, in the fragments). Box plots are drawn using the same colour coding as the main scatter plot (see Fig. 3 legend for details).

A plot of hydrophobicity versus region length shows that a single length threshold effectively segregates Ordered regions from the three other parsed subsets, which are intermingled (81% discrimination of Ordered set, >85% for other three sets on the other side of the line Fig. 4A). Finally, an almost horizontal boundary line was found to discriminate effectively the Ordered and Other-Disordered regions (Fig. 4B), with the Ordered set pulling the FB regions with them (93% correct discrimination ordered, 62% FB regions), and the Other-Disordered set pulling the Disordered-around-FB regions with them (85% Disordered, 82% disordered around FB regions).

Thus, ordered regions are distinguished from the other region types by their hydrophobicity and length, whereas more segregation of Ordered along with FB regions (versus Disordered-around-FB along with Other-Disordered regions) is achieved when conservation is considered.

### Further analysis of compositional differences between the four parsed subsets

**Figure 5:**
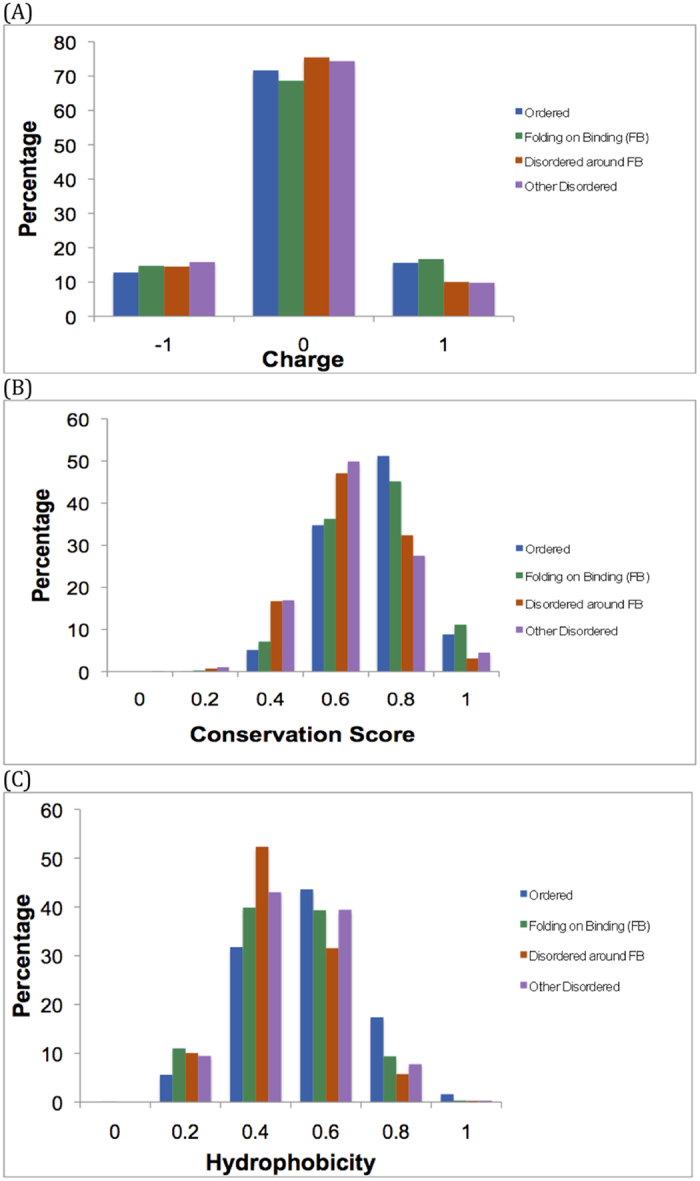
Trends in composition and conservation for the four parsed region types. (**A**) Histogram of charge for the total set of residues for the four subsets. The colour scheme is: Ordered, blue (total = 17868); Other-Disordered, red (2040); FB, green (3205); Disordered-around-FB, orange (2936). Percentages are shown. (**B**) Histogram of hydrophobicity for the total set of residues for the four subsets. The colour scheme is the same as part (**A**). (**C**) Histogram of conservation score for the total set of residues for the four subsets. The colour scheme is the same as part (**A**).

The distribution of hydrophobicity and net charge for the populations of residues in the four parsed subsets (shown in Fig. 5A,B) was analysed for significant differences (Tables 1, 2, 3, 4, [Table t2], [Table t3], [Table t3], [Table t3], [Table t4]). This analysis includes the data for shorter sequence tracts (<20 residues in length).

In composite, the results for hydrophobicity (Tables 1 and 2) indicate the following significant trend:


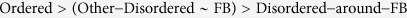


Thus, Disordered-around-FB regions are distinctly the most hydrophilic parsed subset, with FB regions, in general, approximately as hydrophobic as Other-Disordered regions in the same sequences. It has been observed previously that the interfaces of proteins that undergo disorder to order transition are more hydrophobic^25^,^26^, as is generally observed in protein-protein interactions^27^. However, It has also been suggested that the polar and charged amino acids present in FB proteins play a major role in interacting with the partner molecules^28^, thus leading to overall hydrophobicity in FB regions that is here indistinguishable from other disordered tracts; however, the Disordered-around-FB regions are clearly distinct in composition to the FB regions.

The total net charge of each of the four datasets was calculated at pH 7 (Fig. 5A). In composite, the results for net charge (Tables 3 and 4) indicate a significant trend, summarized by the following inequality:


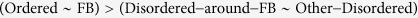


**Figure 6:**
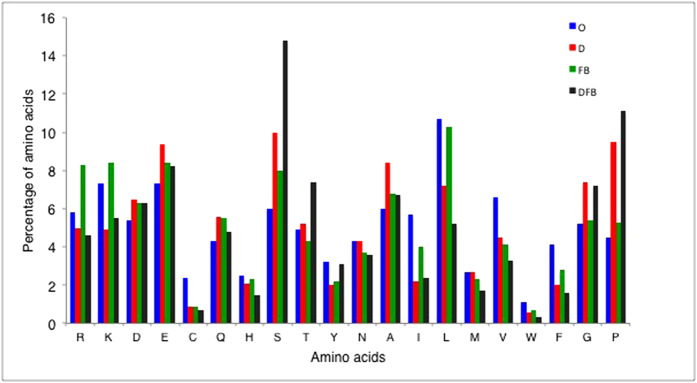
Comparison of the overall amino-acid composition of the four region types. The four subsets are labelled O for Ordered, D for Other-Disordered, FB for Folding-on-binding and DFB for Disordered-around-FB.

Thus, regions that can be structured (Ordered and FB) have overall positive charge, whereas the other sets have negative charge overall. If we examine the prevalences of the twenty amino acids in the four subsets, there are some distinctive trends for each subset (Fig. 6); the Disordered-around-FB regions have a pronounced preference for T, S, G and P, with the Other-Disordered regions having a similar, less pronounced preference for S, G and P. Glycine and proline residues control the flexibility of the polypeptide chain, and so areas rich in these residues may be designed to bend or deform in specific ways.

### Complex pattern of sequence conservation in FB-containing proteins

The distribution of conservation scores (shown in Fig. 5C) was analysed for significant trends (Tables 5 and 6). In composite, we get the following overall tendency for conservation:


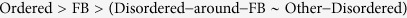


Thus, FB regions are distinctly a highly conserved set, but not as highly conserved as the Ordered set. The Disordered-around-FB and Other-Disordered regions are the most evolutionarily variable (Tables 5 and 6).

### Sampling analysis of parsed subsets

We also analysed the parsed FB subset as a sample of larger total ordered and disordered sets (Table 7). We examined the FB set as a sample of the total ordered regions (Ordered + FB), and also as a sample of the total disordered regions (FB + Disordered-around-FB + Other-Disordered). The results are in agreement with the analyses performed above, with the FB regions being very distinctive among the total disordered set for conservation (<0.1% of the random samples are more conserved) and net charge (<0.1% are more positively charged), and for hydrophobicity in the total ordered set (<0.1% are less hydrophobic).

### A possible guidance mechanism during folding-on-binding

FB regions have high conservation and slight net positive charge, with contiguous disordered regions having low conservation and slight net negative charge and excessive hydrophilicity. Indeed, the Disordered-around-FB regions are excessively hydrophilic compared to the Other-Disordered regions. It is interesting that these results parallel analyses of conserved areas in protein-protein interfaces, which tend to be more hydrophobic than non-conserved parts^27^.

Our results suggest a possible guidance mechanism for FB regions, wherein excessively hydrophilic Disordered-around-FB regions steer the FB towards the binding site of its interaction partner, by lessening the occurrence of off-target interactions, and thus facilitating the folding-on-binding^29^,^30^,^31^. Such an electrostatic steering mechanism has been shown experimentally and simulationally for the binding of the cell cycle regulator p27 to cyclin A^32^,^33^. The positive charge in the FB region is likely due to the charge character of the binding partners, or specific functional design. Indeed, fourteen of the FB regions analysed are for binding DNA/RNA (which are negatively charged), and a further eleven FB regions are nuclear localization signals, which are positively charged for their specific function. We performed enrichment analysis of Gene ontology molecular function categories, using GOrilla^34^. Indeed, the proteins with FB regions are significantly enriched for nucleic acid binding (GO:0003676, corrected P-value = 0.0074) and DNA binding (GO:0003677, corrected P-value = 0.018, using a non-redundant DisProt set as background population), which is consistent with the positive charge of the FB regions. It has been previously shown that the charge in disordered regions correlates with molecular function^35^.

### Concluding remarks

We performed a bioinformatical parsing of folding-on-binding proteins into four distinct region types: Ordered, folding-on-binding (FB), Disordered-around-FB, and Other-Disordered. From a convergence of three separate analyses (treating the sets as fragments, as populations of residues and as samples of fragments from populations), we observe that compositionally, the Ordered regions segregate as more hydrophobic than the three other region types, but that in terms of conservation, the Ordered and FB regions tend to group together and the Disordered-around-FB and Other-Disordered regions with each other, although there is still some lesser significant difference between the Ordered and FB sets. We described how our results point towards a possible compositionally-based steering mechanism of FB region folding-on-binding. Further experimental and simulational work is required to investigate this hypothesis.

## Methods

### Data sets

Human experimentally-verified intrinsically disordered protein sequences were retrieved from the IDEAL (Intrinsically disordered proteins with extensive annotation and literature) database^36^,^37^ (sequences retrieved in August 2014). This gave us a total of 99 human intrinsically disordered proteins with FB regions. For some analysis we also used a data set of 134 disordered proteins from the DisProt (Database of Protein disorder) release 6.02^38^. These data sets were reduced for sequence redundancy (at 40% sequence identity level) using the CD-HIT tool^39^. To make multiple sequence alignments, orthologs of these human proteins in other vertebrates were obtained from the Ensembl database^40^.

### Multiple sequence alignments

Multiple sequence alignments (MSAs) of human intrinsically disordered proteins along with their orthologs from other vertebrates were generated using MUSCLE v3.8.31^41^.

### Conservation analysis of the aligned sequences

The position-specific conservation of the aligned protein sequences was calculated using the AL2CO program^42^. This program was used to calculate a conservation index for each aligned position of the human proteins in the MUSCLE multiple sequence alignments. In AL2CO, the amino acid frequencies at each position are estimated and the conservation index is calculated from these frequencies. The entropy-based method of AL2CO was used to calculate the conservation index. This uses sequence information entropy, and calculates the frequency of amino acids by grouping the amino acids with similar physicochemical properties. We think this is suitable for analyzing intrinsically disordered regions, since they are compositionally defined regions of protein sequences.

### Hydrophobicity and Charge calculation

The hydrophobicity of the aligned sequences in each protein was calculated using the Kyte & Doolittle hydrophobicity scale with a window size of 5^43^. The net charge at pH 7.0 was also calculated by adding up total numbers of positively and negatively charged residues^18^. The absolute value (*i.e.,* the total ‘chargedness’) was also calculated by making all negative values positive (this is presented in Fig. 3A).

## Additional Information

**How to cite this article**: Narasumani, M. and Harrison, P. M. Bioinformatical parsing of folding-on-binding proteins reveals their compositional and evolutionary sequence design. *Sci. Rep.* **5**, 18586; doi: 10.1038/srep18586 (2015).

## Supplementary Material

Supplementary Information

## Figures and Tables

**Table 1:** Comparison of the hydrophobicities of the parsed subsets.

Datasets	P-value*
Ordered vs Other-Disordered	<0.0001
Ordered vs FB	<0.0001
Ordered vs Disordered-around-FB	<0.0001
Other-Disordered vs FB	NS†
Other-Disordered vs Disordered-around-FB	<0.0001
FB vs Disordered-around-FB	<0.0001

^*^P-values for Wilcoxon ranked sum test.

^†^Not significant.

**Table 2 t2:** Mean hydrophobicity values.

Subset	Mean*
Ordered	−0.3219 (±1.373)
Other-Disordered	−0.867 (±1.278)
FB	−0.834 (±1.326)
Disordered-around-FB	−1.026 (±1.178)

^*^Sample sizes: 17869 (Ordered), 2036 (Other-Disordered), 3201 (FB), 2932 (Disordered-around-FB).

**Table 3 t3:** Comparison of the net charges of the parsed subsets.

Datasets	P-value*
Ordered vs Other-Disordered	<0.0001
Ordered vs FB	NS†
Ordered vs Disordered-around-FB	<0.0001
Other-Disordered vs FB	<0.0001
Other-Disordered vs Disordered-around-FB	NS†
FB vs Disordered-around-FB	<0.0001

^*^P-values for Wilcoxon ranked sum test.

^†^Not significant.

**Table 4 t4:** Mean net-charge values.

Dataset	Mean*
Ordered	0.004 (±0.508)
Other-Disordered	−0.060 (±0.502)
FB	0.020 (±0.559)
Disordered-around-FB	−0.045 (±0.493)

^*^Sample sizes as in Table 2.

**Table 5:** Comparison of the conservation scores of the parsed subsets.

Datasets	P-value*
Ordered vs Other-Disordered	<0.0001
Ordered vs FB	0.031
Ordered vs Disordered-around-FB	<0.0001
Other-Disordered vs FB	<0.0001
Other-Disordered vs Disordered-around-FB	NS†
FB vs Disordered-around-FB	<0.0001

^*^P-values for Wilcoxon ranked sum test.

^†^Not significant.

**Table 6:** Mean conservation score values.

Dataset	Mean*
Ordered	0.278(±0.916)
Other-Disordered	−0.368(±1.050)
FB	0.234(±1.021)
Disordered-around-FB	−0.310(±0.986)

^*^Sample sizes as in Table 2.

**Table 7:** FB set as sample of total ordered and total disordered sets.

Sampling*	Ranking of means of each quantity for original set in list of samples**
Conservation	
FB in total ordered	21.6 percentile
FB in total disordered	99.9 percentile
Hydrophobicity	
FB in total ordered	0.1 percentile
FB in total disordered	87.8 percentile
Charge	
FB in total ordered	89.5 percentile
FB in total disordered	99.9 percentile

^*^Total ordered = Ordered + FB; total disordered = Disordered-around-FB + FB + Other-Disordered.

^**^10,000 samples of the same distribution of region lengths as observed for the FB set were taken from each total population of ordered and disordered regions. The ranking for the mean value of the original FB subset in the list of samples is expressed as a percentile, i.e. at 5 percentile, 5% of the samples are less conserved, hydrophobic or positively charged.
