# Supplementary material for: Bioinformatical parsing of folding-on-binding proteins reveals their compositional and evolutionary sequence design
Source: Sci Rep. 2015 Dec 18;5:18586. doi: 10.1038/srep18586 (PMC4683461; doi:10.1038/srep18586)
Supplement: Supplementary Information [file srep18586-s1.pdf]

Bioinformatical parsing of folding-on-binding proteins reveals their compositional and evolutionary sequence design

Mohanalakshmi Narasumani[1] and Paul M Harrison[1] *
[1]Department of Biology, McGill University, Montreal, QC, Canada.

Supplementary Table1. List of human proteins with FB regions retrieved from IDEAL database

IDEAL ID[1]	UniProt Entry[2]	Protein names[3]	Length[4]
IID00001	O00482	Nuclear receptor subfamily 5 group A member 2 (Alpha-1-fetoprotein transcription factor) (B1-binding factor) (hB1F) (CYP7A promoter-binding factor) (Hepatocytic transcription factor) (Liver receptor homolog 1) (LRH-1)	541
IID00232	O00571	ATP-dependent RNA helicase DDX3X (EC 3.6.4.13) (DEAD box protein 3, X-chromosomal) (DEAD box, X isoform) (Helicase-like protein 2) (HLP2)	662
IID00134	O14745	Na(+)/H(+) exchange regulatory cofactor NHE-RF1 (NHERF-1) (Ezrin-radixin-moesin-binding phosphoprotein 50) (EBP50) (Regulatory cofactor of Na(+)/H(+) exchanger) (Sodium-hydrogen exchanger regulatory factor 1) (Solute carrier family 9 isoform A3 regulatory factor 1)	358
IID00006	O15162	Phospholipid scramblase 1 (PL scramblase 1) (Ca(2+)-dependent phospholipid scramblase 1) (Erythrocyte phospholipid scramblase) (MmTRA1b)	318
IID00007	O15169	Axin-1 (Axis inhibition protein 1) (hAxin)	862
IID00185	O15234	Protein CASC3 (Cancer susceptibility candidate gene 3 protein) (Metastatic lymph node gene 51 protein) (MLN 51) (Protein barentsz) (Btz)	703
IID00189	O75376	Nuclear receptor corepressor 1 (N-CoR) (N-CoR1)	2440
IID00115	O95149	Snurportin-1 (RNA U transporter 1)	360
IID00112	O95405	Zinc finger FYVE domain-containing protein 9 (Mothers against decapentaplegic homolog-interacting protein) (Madh-interacting protein) (Novel serine protease) (NSP) (Receptor activation anchor) (hSARA) (Smad anchor for receptor activation)	1425
IID00011	O95718	Steroid hormone receptor ERR2 (ERR beta-2) (Estrogen receptor-like 2) (Estrogen-related receptor	508

		beta) (ERR-beta) (Nuclear receptor subfamily 3 group B member 2)	
IID00262	P00533	Epidermal growth factor receptor (EC 2.7.10.1) (Proto-oncogene c-ErbB-1) (Receptor tyrosine-protein kinase erbB-1)	1210
IID00012	P01106	Myc proto-oncogene protein (Class E basic helix-loop-helix protein 39) (bHLHe39) (Proto-oncogene c-Myc) (Transcription factor p64)	439
IID00013	P03372	Estrogen receptor (ER) (ER-alpha) (Estradiol receptor) (Nuclear receptor subfamily 3 group A member 1)	595
IID00137	P04083	Annexin A1 (Annexin I) (Annexin-1) (Calpactin II) (Calpactin-2) (Chromobindin-9) (Lipocortin I) (Phospholipase A2 inhibitory protein) (p35)	346
IID00015	P04637	Cellular tumor antigen p53 (Antigen NY-CO-13) (Phosphoprotein p53) (Tumor suppressor p53)	393
IID00017	P06400	Retinoblastoma-associated protein (p105-Rb) (pRb) (Rb) (pp110)	928
IID00119	P09651	Heterogeneous nuclear ribonucleoprotein A1 (hnRNP A1) (Helix-destabilizing protein) (Single-strand RNA-binding protein) (hnRNP core protein A1) [Cleaved into: Heterogeneous nuclear ribonucleoprotein A1, N-terminally processed]	372
IID00194	P12272	Parathyroid hormone-related protein (PTH-rP) (PTHrP) (Parathyroid hormone-like protein) (PLP) [Cleaved into: PTHrP[1-36]; PTHrP[38-94]; Osteostatin (PTHrP[107-139])]	177
IID00023	P12956	X-ray repair cross-complementing protein 6 (EC 3.6.4.-) (EC 4.2.99.-) (5'-deoxyribose-5-phosphate lyase Ku70) (5'-dRP lyase Ku70) (70 kDa subunit of Ku antigen) (ATP-dependent DNA helicase 2 subunit 1) (ATP-dependent DNA helicase II 70 kDa subunit) (CTC box-binding factor 75 kDa subunit) (CTC75) (CTCBF) (DNA repair protein XRCC6) (Lupus Ku autoantigen protein p70) (Ku70) (Thyroid-lupus autoantigen) (TLAA) (X-ray repair complementing defective repair in Chinese hamster cells 6)	609
IID00024	P13010	X-ray repair cross-complementing protein 5 (EC 3.6.4.-) (86 kDa subunit of Ku antigen) (ATP-dependent DNA helicase 2 subunit 2) (ATP-dependent DNA helicase II 80 kDa subunit) (CTC box-binding factor 85 kDa subunit) (CTC85) (CTCBF) (DNA repair protein XRCC5) (Ku80) (Ku86) (Lupus Ku autoantigen protein p86) (Nuclear factor IV)	732

		(Thyroid-lupus autoantigen) (TLAA) (X-ray repair complementing defective repair in Chinese hamster cells 5 (double-strand-break rejoining))	
IID00145	P17096	High mobility group protein HMG-I/HMG-Y (HMG-I(Y)) (High mobility group AT-hook protein 1) (High mobility group protein A1) (High mobility group protein R)	107
IID00121	P22415	Upstream stimulatory factor 1 (Class B basic helix-loop-helix protein 11) (bHLHb11) (Major late transcription factor 1)	310
IID00196	P23025	DNA repair protein complementing XP-A cells (Xeroderma pigmentosum group A-complementing protein)	273
IID00126	P24928	DNA-directed RNA polymerase II subunit RPB1 (RNA polymerase II subunit B1) (EC 2.7.7.6) (DNA-directed RNA polymerase II subunit A) (DNA-directed RNA polymerase III largest subunit) (RNA-directed RNA polymerase II subunit RPB1) (EC 2.7.7.48)	1970
IID00035	P25054	Adenomatous polyposis coli protein (Protein APC) (Deleted in polyposis 2.5)	2843
IID00148	P28324	ETS domain-containing protein Elk-4 (Serum response factor accessory protein 1) (SAP-1) (SRF accessory protein 1)	431
IID00130	P28749	Retinoblastoma-like protein 1 (107 kDa retinoblastoma-associated protein) (p107) (pRb1)	1068
IID00037	P31751	RAC-beta serine/threonine-protein kinase (EC 2.7.11.1) (Protein kinase Akt-2) (Protein kinase B beta) (PKB beta) (RAC-PK-beta)	481
IID00039	P35222	Catenin beta-1 (Beta-catenin)	781
IID00043	P38936	Cyclin-dependent kinase inhibitor 1 (CDK-interacting protein 1) (Melanoma differentiation-associated protein 6) (MDA-6) (p21)	164
IID00044	P39748	Flap endonuclease 1 (FEN-1) (EC 3.1.-.-) (DNase IV) (Flap structure-specific endonuclease 1) (Maturation factor 1) (MF1) (hFEN-1)	380
IID00047	P42226	Signal transducer and activator of transcription 6 (IL-4 Stat)	847
IID00269	P42768	Wiskott-Aldrich syndrome protein (WASp)	502
IID00049	P46527	Cyclin-dependent kinase inhibitor 1B (Cyclin-dependent kinase inhibitor p27) (p27Kip1)	198
IID00050	P48552	Nuclear receptor-interacting protein 1 (Nuclear factor RIP140) (Receptor-interacting protein 140)	1158
IID00271	P49137	MAP kinase-activated protein kinase 2 (MAPK-activated protein kinase 2) (MAPKAP kinase 2) (MAPKAP-K2) (MAPKAPK-2) (MK-2) (MK2) (EC	400

		2.7.11.1)	
IID00151	P49792	E3 SUMO-protein ligase RanBP2 (EC 6.3.2.-) (358 kDa nucleoporin) (Nuclear pore complex protein Nup358) (Nucleoporin Nup358) (Ran-binding protein 2) (RanBP2) (p270)	3224
IID00052	P49841	Glycogen synthase kinase-3 beta (GSK-3 beta) (EC 2.7.11.26) (Serine/threonine-protein kinase GSK3B) (EC 2.7.11.1)	420
IID00237	P51587	Breast cancer type 2 susceptibility protein (Fanconi anemia group D1 protein)	3418
IID00152	P52272	Heterogeneous nuclear ribonucleoprotein M (hnRNP M)	730
IID00153	P52292	Importin subunit alpha-1 (Karyopherin subunit alpha-2) (RAG cohort protein 1) (SRP1-alpha)	529
IID00238	P52298	Nuclear cap-binding protein subunit 2 (20 kDa nuclear cap-binding protein) (Cell proliferation-inducing gene 55 protein) (NCBP 20 kDa subunit) (CBP20) (NCBP-interacting protein 1) (NIP1)	156
IID00273	P52565	Rho GDP-dissociation inhibitor 1 (Rho GDI 1) (Rho-GDI alpha)	204
IID00053	P52630	Signal transducer and activator of transcription 2 (p113)	851
IID00122	P52907	F-actin-capping protein subunit alpha-1 (CapZ alpha-1)	286
IID00200	P53350	Serine/threonine-protein kinase PLK1 (EC 2.7.11.21) (Polo-like kinase 1) (PLK-1) (Serine/threonine-protein kinase 13) (STPK13)	603
IID00154	P54198	Protein HIRA (TUP1-like enhancer of split protein 1)	1017
IID00155	P54274	Telomeric repeat-binding factor 1 (NIMA-interacting protein 2) (TTAGGG repeat-binding factor 1) (Telomeric protein Pin2/TRF1)	439
IID00201	P55072	Transitional endoplasmic reticulum ATPase (TER ATPase) (EC 3.6.4.6) (15S Mg(2+)-ATPase p97 subunit) (Valosin-containing protein) (VCP)	806
IID00274	P61586	Transforming protein RhoA (Rho cDNA clone 12) (h12)	193
IID00058	P62805	Histone H4	103
IID00162	P62826	GTP-binding nuclear protein Ran (Androgen receptor-associated protein 24) (GTPase Ran) (Ras-like protein TC4) (Ras-related nuclear protein)	216
IID00062	P68431	Histone H3.1 (Histone H3/a) (Histone H3/b) (Histone H3/c) (Histone H3/d) (Histone H3/f) (Histone H3/h) (Histone H3/i) (Histone H3/j) (Histone H3/k) (Histone H3/l)	136

IID00163	Q00987	E3 ubiquitin-protein ligase Mdm2 (EC 6.3.2.-) (Double minute 2 protein) (Hdm2) (Oncoprotein Mdm2) (p53-binding protein Mdm2)	491
IID00064	Q01094	Transcription factor E2F1 (E2F-1) (PBR3) (Retinoblastoma-associated protein 1) (RBAP-1) (Retinoblastoma-binding protein 3) (RBBP-3) (pRB-binding protein E2F-1)	437
IID00206	Q01658	Protein Dr1 (Down-regulator of transcription 1) (Negative cofactor 2-beta) (NC2-beta) (TATA-binding protein-associated phosphoprotein)	176
IID00066	Q02156	Protein kinase C epsilon type (EC 2.7.11.13) (nPKC-epsilon)	737
IID00207	Q04206	Transcription factor p65 (Nuclear factor NF-kappa-B p65 subunit) (Nuclear factor of kappa light polypeptide gene enhancer in B-cells 3)	551
IID00209	Q05086	Ubiquitin-protein ligase E3A (EC 6.3.2.-) (E6AP ubiquitin-protein ligase) (Human papillomavirus E6-associated protein) (Oncogenic protein-associated protein E6-AP) (Renal carcinoma antigen NY-REN-54)	875
IID00165	Q05195	Max dimerization protein 1 (Max dimerizer 1) (Protein MAD)	221
IID00276	Q07912	Activated CDC42 kinase 1 (ACK-1) (EC 2.7.10.2) (EC 2.7.11.1) (Tyrosine kinase non-receptor protein 2)	1038
IID00069	Q08209	Serine/threonine-protein phosphatase 2B catalytic subunit alpha isoform (EC 3.1.3.16) (CAM-PRP catalytic subunit) (Calmodulin-dependent calcineurin A subunit alpha isoform)	521
IID00167	Q09161	Nuclear cap-binding protein subunit 1 (80 kDa nuclear cap-binding protein) (CBP80) (NCBP 80 kDa subunit)	790
IID00169	Q13127	RE1-silencing transcription factor (Neural-restrictive silencer factor) (X2 box repressor)	1097
IID00277	Q13153	Serine/threonine-protein kinase PAK 1 (EC 2.7.11.1) (Alpha-PAK) (p21-activated kinase 1) (PAK-1) (p65-PAK)	545
IID00170	Q13541	Eukaryotic translation initiation factor 4E-binding protein 1 (4E-BP1) (eIF4E-binding protein 1) (Phosphorylated heat- and acid-stable protein regulated by insulin 1) (PHAS-I)	118
IID00074	Q13772	Nuclear receptor coactivator 4 (NCoA-4) (Androgen receptor coactivator 70 kDa protein) (70 kDa AR-activator) (70 kDa androgen receptor coactivator) (Androgen receptor-associated protein of 70 kDa) (Ret-activating protein ELE1)	614

IID00075	Q14103	Heterogeneous nuclear ribonucleoprotein D0 (hnRNP D0) (AU-rich element RNA-binding protein 1)	355
IID00080	Q15054	DNA polymerase delta subunit 3 (DNA polymerase delta subunit p66)	466
IID00172	Q15208	Serine/threonine-protein kinase 38 (EC 2.7.11.1) (NDR1 protein kinase) (Nuclear Dbf2-related kinase 1)	465
IID00081	Q15466	Nuclear receptor subfamily 0 group B member 2 (Orphan nuclear receptor SHP) (Small heterodimer partner)	257
IID00082	Q15596	Nuclear receptor coactivator 2 (NCoA-2) (Class E basic helix-loop-helix protein 75) (bHLHe75) (Transcriptional intermediary factor 2) (hTIF2)	1464
IID00173	Q15648	Mediator of RNA polymerase II transcription subunit 1 (Activator-recruited cofactor 205 kDa component) (ARC205) (Mediator complex subunit 1) (Peroxisome proliferator-activated receptor-binding protein) (PBP) (PPAR-binding protein) (Thyroid hormone receptor-associated protein complex 220 kDa component) (Trap220) (Thyroid receptor-interacting protein 2) (TR-interacting protein 2) (TRIP-2) (Vitamin D receptor-interacting protein complex component DRIP205) (p53 regulatory protein RB18A)	1581
IID00083	Q15788	Nuclear receptor coactivator 1 (NCoA-1) (EC 2.3.1.48) (Class E basic helix-loop-helix protein 74) (bHLHe74) (Protein Hin-2) (RIP160) (Renal carcinoma antigen NY-REN-52) (Steroid receptor coactivator 1) (SRC-1)	1441
IID00127	Q15796	Mothers against decapentaplegic homolog 2 (MAD homolog 2) (Mothers against DPP homolog 2) (JV18-1) (Mad-related protein 2) (hMAD-2) (SMAD family member 2) (SMAD 2) (Smad2) (hSMAD2)	467
IID00213	Q16236	Nuclear factor erythroid 2-related factor 2 (NF-E2-related factor 2) (NFE2-related factor 2) (HEBP1) (Nuclear factor, erythroid derived 2, like 2)	605
IID00175	Q16633	POU domain class 2-associating factor 1 (B-cell-specific coactivator OBF-1) (BOB-1) (OCA-B) (OCT-binding factor 1)	256
IID00085	Q16665	Hypoxia-inducible factor 1-alpha (HIF-1-alpha) (HIF1-alpha) (ARNT-interacting protein) (Basic-helix-loop-helix-PAS protein MOP1) (Class E basic helix-loop-helix protein 78) (bHLHe78) (Member of PAS protein 1) (PAS domain-containing protein 8)	826
IID00216	Q6VMQ6	Activating transcription factor 7-interacting protein 1	1270

		(ATF-interacting protein) (ATF-IP) (ATF7-interacting protein) (ATFa-associated modulator) (hAM) (MBD1-containing chromatin-associated factor 1) (P621)	
IID00282	Q86U70	LIM domain-binding protein 1 (LDB-1) (Carboxyl-terminal LIM domain-binding protein 2) (CLIM-2) (LIM domain-binding factor CLIM2) (hLdb1) (Nuclear LIM interactor)	411
IID00220	Q92585	Mastermind-like protein 1 (Mam-1)	1016
IID00092	Q92793	CREB-binding protein (EC 2.3.1.48)	2442
IID00093	Q92837	Proto-oncogene FRAT1 (Frequently rearranged in advanced T-cell lymphomas 1) (FRAT-1)	279
IID00177	Q96IZ0	PRKC apoptosis WT1 regulator protein (Prostate apoptosis response 4 protein) (Par-4)	340
IID00222	Q99081	Transcription factor 12 (TCF-12) (Class B basic helix-loop-helix protein 20) (bHLHb20) (DNA-binding protein HTF4) (E-box-binding protein) (Transcription factor HTF-4)	682
IID00179	Q99967	Cbp/p300-interacting transactivator 2 (MSG-related protein 1) (MRG-1) (P35srj)	270
IID00252	Q9HAU5	Regulator of nonsense transcripts 2 (Nonsense mRNA reducing factor 2) (Up-frameshift suppressor 2 homolog) (hUpf2)	1272
IID00125	Q9HB71	Calcyclin-binding protein (CacyBP) (hCacyBP) (S100A6-binding protein) (Siah-interacting protein)	228
IID00253	Q9HD42	Charged multivesicular body protein 1a (Chromatin-modifying protein 1a) (CHMP1a) (Vacuolar protein sorting-associated protein 46-1) (Vps46-1) (hVps46-1)	196
IID00100	Q9NQB0	Transcription factor 7-like 2 (HMG box transcription factor 4) (T-cell-specific transcription factor 4) (T-cell factor 4) (TCF-4) (hTCF-4)	619
IID00102	Q9NSA3	Beta-catenin-interacting protein 1 (Inhibitor of beta-catenin and Tcf-4)	81
IID00103	Q9UBK2	Peroxisome proliferator-activated receptor gamma coactivator 1-alpha (PGC-1-alpha) (PPAR-gamma coactivator 1-alpha) (PPARGC-1-alpha) (Ligand effect modulator 6)	798
IID00104	Q9UBT2	SUMO-activating enzyme subunit 2 (EC 6.3.2.-) (Anthracycline-associated resistance ARX) (Ubiquitin-like 1-activating enzyme E1B) (Ubiquitin-like modifier-activating enzyme 2)	640
IID00105	Q9UBT6	DNA polymerase kappa (EC 2.7.7.7) (DINB protein) (DINP)	870
IID00225	Q9UBU9	Nuclear RNA export factor 1 (Tip-associated protein)	619

		(Tip-associating protein) (mRNA export factor TAP)	
IID00226	Q9UKV5	E3 ubiquitin-protein ligase AMFR (EC 6.3.2.-) (Autocrine motility factor receptor) (AMF receptor) (RING finger protein 45) (gp78)	643
IID00230	Q9Y3E7	Charged multivesicular body protein 3 (Chromatin-modifying protein 3) (Neuroendocrine differentiation factor) (Vacuolar protein sorting-associated protein 24) (hVps24)	222
IID00117	Q9Y5B0	RNA polymerase II subunit A C-terminal domain phosphatase (EC 3.1.3.16) (TFIIF-associating CTD phosphatase)	961
IID00109	Q9Y618	Nuclear receptor corepressor 2 (N-CoR2) (CTG repeat protein 26) (SMAP270) (Silencing mediator of retinoic acid and thyroid hormone receptor) (SMRT) (T3 receptor-associating factor) (TRAC) (Thyroid-, retinoic-acid-receptor-associated corepressor)	2525

[1] Protein identification number in IDEAL database.

[2] protein identification number in UniProt database.

[3] Protein name given in IDEAL database.

[4] Total length of each protein given in IDEAL database.
